# Characterization of Titanium Corrosion and Its Association With Peri-Implantitis Using Energy Dispersive X-ray Spectroscopy: A Case Report

**DOI:** 10.7759/cureus.60533

**Published:** 2024-05-17

**Authors:** Abhinav Atchuta, Ajay Reddy, Surabhi Bhadauriya, Mounika Beeravolu, Sanjay Vasudevan

**Affiliations:** 1 Periodontology, Army College of Dental Sciences, Secunderabad, IND; 2 Periodontology, Kamineni Institute of Dental Sciences, Nalgonda, IND

**Keywords:** dental implant failure, dental titanium implant, sem edx, peri-implantitis, corrosion

## Abstract

Dental implant corrosion is now being recognized as a contributing factor in the onset and advancement of peri-implantitis, posing significant challenges to both the durability of implants and the well-being of patients. The dissemination of titanium microparticles due to corrosion raises concerns about plausible toxicity and biological effects, especially for patients with long-standing implant prostheses. This case report focuses on the release of titanium particles in the peri-implant mucosa due to corrosion and its association with peri-implantitis. It emphasizes the critical need for strategies to minimize corrosion and alleviate its detrimental effects in order to optimize patient outcomes in the field of implant dentistry. Additionally, there is a call for research into the increasing biochemical effects of these microparticles on oral soft tissues surrounding metallic implants to enhance the longevity and clinical outcomes of implants.

## Introduction

Recently, dental implants have emerged as the key component of oral rehabilitation. Dental implants, made of titanium and titanium alloys, are biomaterials used to regain lost chewing function and aesthetics in various completely or partially edentulous conditions. They should be nonallergic, sterilizable, biocompatible, and resistant to corrosion and occlusal loads [1]. Titanium is considered a bioinert or bio-tolerant material. It has a strong oxidizing surface that rapidly forms a titanium dioxide (TiO2) layer when exposed to fluids or air. This makes titanium extremely resistant to corrosion and significantly reduces, although does not completely prevent, ion release. The passive oxide layer in titanium implants comprises either anatase and rutile or just anatase. It causes passivation of the metal, which determines the implant’s biological response and level of biocompatibility [2]. Surface treatments of implant alloys can considerably reduce the release of the microparticles [3].

Corrosion is the breakdown of the metal surface caused by the surrounding medium (electrochemical attack), resulting in the release of ionized particles into the surrounding environment [4]. The saliva in the oral cavity consists of metallic salts that act as polyelectrolytes, leading to various types of electrochemical corrosion. The most common type of corrosion found in dental implants is galvanic corrosion [5].

Apart from electrochemical corrosion, mechanical wear of implant surfaces caused by friction during insertion, connecting abutments, and removing failing implants could all be potential causes of the presence of these microparticles in peri-implant tissues [6]. Titanium microparticles in the peri-implant mucosa can initiate a foreign body response in the host, which depends on their quantity and physicochemical properties, as well as the host immunity. Pro-inflammatory cytokines, chemokines, and anti-inflammatory cytokines may influence the progression into peri-implantitis. Imbalanced cytokine release can hinder inflammation resolution and contribute to alveolar bone loss [7].

Despite having high success rates, peri-implant infections (peri-mucositis or peri-implantitis), excessive occlusal loads, and implant-dependent factors cause implant failures in the long term. A small number of cases have reported the probability of implant failures due to titanium hypersensitivity [8].

Difficulty in accurately identifying trace elements within tissues can lead to misunderstandings regarding the underlying cause of implant failure. Energy dispersive X-ray spectroscopy (EDX) is required to detect the elements present in a material by using a scanning electron microscope (SEM). It enables many elements in solid samples to be measured rapidly, cheaply, and accurately. It was reported to be successful in determining the chemical composition of soft tissues around implants [9].

This case report includes a classic clinical case of peri-implantitis in which the accumulation of titanium particles was determined in the peri-implant tissue with SEM-EDX analysis.

## Case presentation

History and clinical examination

**Figure 1:**
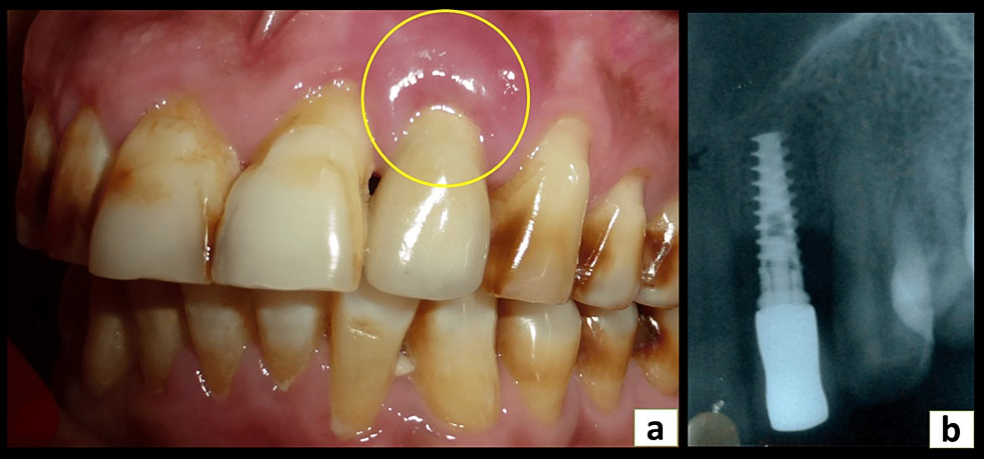
A 62-year-old patient with the implant in relation to the maxillary left lateral incisor (a) Preoperative photograph showing inflamed peri-implant mucosa (yellow circle) in relation to the maxillary left lateral incisor. (b) Preoperative radiograph presenting horizontal bone loss surrounding the implant.

A 62-year-old male patient visited the periodontology department with a chief complaint of gradual, continuous, gnawing pain and discomfort in the upper left front tooth region for two months. The upper left lateral incisor had been replaced with a dental implant 15 months earlier. The patient was systemically healthy and a non-smoker. Clinical examination of the site revealed diffuse bluish-pink gingiva, spontaneous bleeding on probing, a pocket depth of 7 mm, and a lack of mobility, as well as gingival recession on adjacent teeth (Figure 1a). The oral hygiene of the patient was fair, with an Oral Hygiene Index-Simplified (OHI-S) score of 1.

Radiographic findings

Radiographically, there was evident horizontal bone loss (Schwarz class II bone defect) (Figure 1b).

Provisional diagnosis

Based on the clinical and radiographic findings, the condition was diagnosed as peri-implantitis, and surgical intervention was planned as regenerative therapy.

Surgical intervention

Under adequate local anesthesia (2% lignocaine), a sulcular incision was made using a No. 15 BP blade, involving one tooth on either side of the implant, and a vertical releasing incision was made distal to the canine. A full-thickness flap was elevated, and peri-implant debridement was performed using a plastic curette. Surface decontamination was done using an air polisher, and chlorhexidine was used for irrigation. The flaps were approximated after filling the defect with a bone graft covered by a membrane using interrupted sutures. After two weeks, exposure of the abutment collar was observed due to soft tissue loss.

SEM-EDX analysis

**Figure 2:**
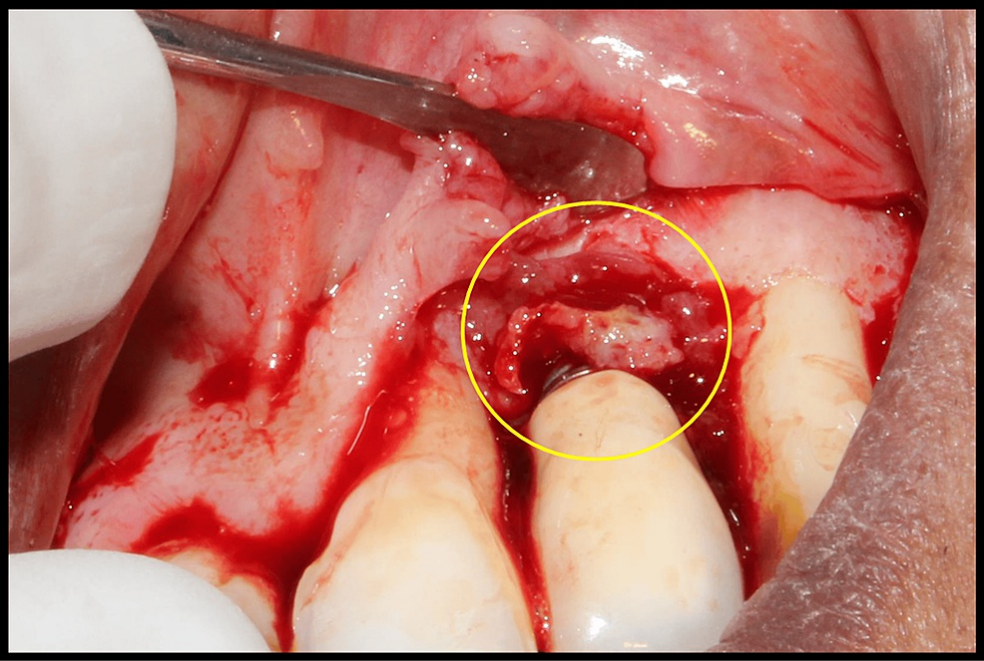
Intraoperative photograph showing granulation tissue in the region of the lateral incisor (yellow circle) that was sent for SEM-EDX analysis EDX, energy dispersive X-ray spectroscopy; SEM, scanning electron microscope

The peri-implant tissue obtained after debridement was embedded in formalin and sent for SEM-EDX for the detection of titanium particles in the peri-implant tissue (Figure 2). This analysis aimed to characterize the morphology and elemental composition of the foreign material. The specimens were evaluated under conditions suitable for organic, nonconductive samples using a low-vacuum EDX mode at various magnifications to pinpoint areas for detailed analysis.

SEM-EDX analysis findings

**Figure 3:**
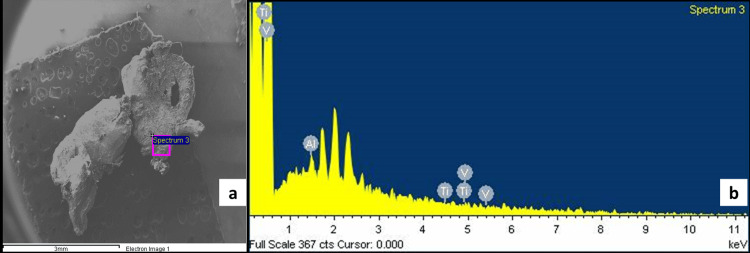
SEM-EDX analysis (a) SEM revealed particles of varying sizes. (b) Analysis using EDX confirmed the existence of titanium particles. EDX, energy dispersive X-ray spectroscopy; SEM, scanning electron microscope

The biopsy tissue embedded in paraffin revealed the presence of 52.42% carbon (C) and 45.73% oxygen (O). Further analysis presented titanium peaks along with traces of aluminum and vanadium. These titanium peaks validate the presence of Ti particles in the peri-implant mucosa (Figure 3a, 3b).

## Discussion

Implant treatment has revolutionized the way we treat tooth loss and serves as a viable alternative to traditional prosthetics. Nonetheless, studies indicate that peri-implantitis can still impact a significant percentage of implants, ranging from 2.7% to 47.1%, despite successful osseointegration [10]. While it is widely acknowledged that peri-implantitis stems from inflammation triggered by bacterial plaque, additional factors like titanium metal particles have been proposed as potential contributors to the condition [11,12].

Research has validated the dissemination of titanium particles into adjacent tissues from titanium implants [6,12,13], yet the exact influence of titanium particles on peri-implantitis remains unclear. Corrosion stands out as one potential factor leading to implant failure following initial success [14,15]. Titanium particles may infiltrate peri-implant tissues as a result of corrosion due to salivary components in the oral environment, microscopic movements between various components of implants, manipulation of the implant surface during instrumentation, or the implant surgical procedure itself [6]. The potential for corrosion and the potent detrimental effects of corrosion by-products in oral tissues are of clinical relevance.

In orthopedic studies, it is firmly established that wear debris significantly contributes to osteolysis and the aseptic loosening of prosthetics [16]. However, in dental research, investigations to substantiate this correlation are still pending.

Recent studies have centered on determining whether titanium particles can trigger foreign body reactions by activating pro-inflammatory cytokines, leading to the loss of peri-implant bone and soft tissue [17]. In a literature review examining the effects of corrosion by-products of titanium implants on peri-implant tissues, Noronha Oliveira et al. documented that titanium particles stimulate osteoclast activity, elevate macrophage presence at the site, and lead to increased mutations in biological cells that were cultured with titanium-based nanoparticles [18]. In 2019, Schwarz et al. discovered that titanium particles impeded the biochemical activity of mesenchymal cells like osteoblasts and fibroblasts [19]. Similarly, a study done in 2017 showed an elevation in pro-inflammatory mediators, such as IL-6, IL-8, and receptor activators of nuclear factor kappa-B ligand, in response to titanium and other metal ions [17]. These mediators are responsible for the initiation and progression of alveolar bone resorption; discrepancies in their release levels may affect the healing of the per-implant mucosa and can lead to alveolar bone destruction [15].

In the present case, the peri-implant tissue was inflamed, and the possible cause of the inflammatory process was evaluated by analysis of this peri-implant tissue. Furthermore, there was a supracrestal bone loss around the implant, which can be explained as metal corrosion that had affected the contact between the implant surface and the alveolar bone. Hence, it can be speculated that the microparticles detected in the biopsy in this case might cause macrophages to fuse into giant cells, triggering a foreign body reaction, or they could degrade into nanoparticles due to corrosion (Figure 3a, 3b).

A few studies indicate that titanium particles have the potential to spread to remote organs and could result in adverse effects such as carcinogenicity, cytotoxicity, metal hypersensitivity, and genotoxicity. The corrosion products of metal implants may act as haptens, triggering a hypersensitivity reaction characterized by the production of pro-inflammatory cytokines and the recruitment of macrophages [7,20].

Although current evidence indicates that increased implant surface corrosion, influenced by factors such as saliva, mechanical wear, acidic environments, and occlusal loads, can lead to foreign body reactions or pro-inflammatory responses resulting in bone loss and peri-implantitis, it is crucial to also consider other contributing factors. These include excessive cement in cement-retained prostheses, poor oral hygiene, and systemic health issues, which are also likely to cause peri-implantitis.

## Conclusions

Corrosion could emerge as a significant contributing factor to the development and progression of peri-implantitis, exerting detrimental effects on peri-implant tissues and host-microbe interactions. Strategies aiming at minimizing corrosion and its adverse effects are necessary for successful implant treatment and optimizing patient outcomes in implant dentistry. SEM-EDX analysis in this case revealed the accumulation of Ti particles in the peri-implant mucosa. The possible toxicity and potential biological complications linked with the released microparticles and ions due to corrosion of the implants may become a concern for patients with dental implants as they remain exposed in the oral environment for a prolonged duration. Advanced research is necessary to confirm the potential long-term adverse effects of the released metal ions on the oral mucosa. Furthermore, the dissemination of ionized Ti particles and their effect on distant organs should be thoroughly investigated.
